# Effects of crystalline lens characteristics on vault after implantable collamer lens implantation

**DOI:** 10.1186/s12886-025-04406-z

**Published:** 2025-10-07

**Authors:** Jieli Wu, Qian Tan, Wang Cai, Ding Lin, Libei Zhao

**Affiliations:** 1 Aier Academy of Ophthalmology, Central South University, Changsha, Hunan China; 2 Changsha Aier Eye Hospital, Changsha, Hunan, China; 3 Xiangya Hospital, Central South University, Changsha, Hunan, China

**Keywords:** Crystalline lens, Vault, Implantable Collamer lens, Myopia, AS-OCT

## Abstract

**Purpose:**

To investigate the association between crystalline lens characteristics and postoperative vault in highly myopic eyes following implantation of Implantable Collamer Lens V4c (ICL V4c; STAAR Surgical).

**Setting:**

Changsha Aier Eye Hospital, Aier Eye Hospital Group, Changsha, China.

**Design:**

Retrospective observational study.

**Methods:**

This retrospective study included 199 eyes from 199 patients who underwent ICL implantation. Crystalline lens Dimensional parameters were quantified using CASIA 2 swept-source anterior segment optical coherence tomography. Based on vault height measured 3 months postoperatively, eyes were classified into low (< 250 µm), optimal (250–750 µm), or high (> 750 µm) vault groups. Group comparisons, Spearman correlation, and multivariate logistic regression analyses were performed to identify factors associated with low vault outcomes.

**Results:**

Significant differences were observed in white-to-white (WTW), anterior chamber depth (ACD), anterior radius of curvature (ARC), posterior radius of curvature (PRC), lens thickness (LT), and decentration among the different vault groups (all *P* < 0.001). Multivariate logistic regression analysis identified a significant negative association between low vault and both ARC and lens tilt, after adjusting for age, gender and spherical equivalent (all *P* < 0.05).

**Conclusions:**

Crystalline Lens parameters play a critical role in determining postoperative vault following ICL implantation. Steeper anterior curvature and greater lens tilt may be anatomic indicators of a predisposition to low postoperative vault outcomes.

## Introduction

Myopia is the most common refractive error globally, with an estimated half of the world’s population expected to be affected by 2050, and nearly 10% anticipated to have high myopia [1]. The prevalence of myopia and high myopia has shown a rapid increase, particularly in young East and Southeast Asians [1, 2]. The Implantable Collamer Lens (ICL; STAAR Surgical, Nidau, Switzerland) with a central hole facilitates the surgical procedure and safely and effectively corrects high myopia, super high myopia, astigmatism, and presbyopia [3–6].

Proper ICL sizing with an appropriate postoperative vault is vital for the success of the procedure. The consensus is that the optimal vault, which is the Distance between the posterior surface of the ICL and the anterior surface of the crystalline lens, should range from 250 µm to 750 µm^7^. Despite the manufacturer's provision of an online calculation for ICL sizing based on the white-to-white (horizontal corneal diameter, WTW) and anterior chamber depth (ACD), a significant proportion of cases still fall outside the optimal vault range [7, 8]. Consequently, many formulas and preoperative anatomic parameters have been proposed to optimise the method of ICL sizing [9–11]. Given the complexity of anterior segment biometrics, predicting the vault and ICL size based on preoperative parameters identified in previous studies remains problematic.

Theoretically, the ICL is positioned anteriorly to the crystalline lens and settles in the ciliary sulcus. However, current study has observed that the shape of the crystalline lens in high myopia tends to vary [12]. To improve the accuracy and precision of vault prediction, variables of the crystalline lens (e.g., crystalline lens rise, lens thickness, lens position, and lens surface curvature radius) have been introduced and are considered to be correlated with the vault [13–15]. The measurement of lens parameters depends directly on images scanned by high-frequency ultrasound biomicroscopy (UBM) and anterior segment optical coherence tomography (AS-OCT). However, UBM is invasive and shows poor reproducibility due to unstable measurement sites. In contrast, AS-OCT is widely used to image the lens and anterior part of the eye, owing to its non-invasiveness and enhanced accuracy. Recently, a new generation of swept-source AS-OCT (CASIA 2, Tomey Corporation, Nagoya, Japan) has been used to assess detailed crystalline lens characteristics in a three-dimensional manner [16]. This method can calculate detailed dimensional parameters related to the crystalline lens, including the anterior and posterior curvatures, the diameter, the decentration, and the tilt of the lens.

The present study intended to describe the lens morphologic features in Different vault at 3 months postoperatively using AS-OCT. Subsequently, we investigated the vault effects of the crystalline lens characteristics in highly myopic eyes following ICL V4c implantation.

## Methods

### Patients

This retrospective study was conducted at Changsha Aier Eye Hospital between July 2021 and March 2023, and was approved by the Ethics Committee of the Changsha Aier Eye Hospital, Aier Eye Hospital Group, China (ID:2021KYYJ004). All procedures were conducted in accordance with the tenets of the Declaration of Helsinki, and informed consent was obtained from all participants before enrolment. A total of 199 eligible eyes from 199 myopic patients who underwent Vision ICL V4c implantation were included in this research. Based on the ICL vault values, the eyes were divided into three subgroups: optimal vault group (OV: 250 µm to 750 µm), low vault group (LV: < 250 µm), and high vault group (HV: > 750 µm). The inclusion criteria were as follows: (1) spherical equivalent (SE) between −6.0 and −18.00 diopters (D); (2) anterior chamber depth (ACD) ≥ 2.8 mm; (3) ICL sizing strictly according to the manufacturer’s recommendations; and (4) corneal endothelial cell density > 2000 cells/mm^2^. The exclusion criteria were refractive astigmatism > −6.0 D, age under 18 years, history of ocular disease, prior ophthalmic surgery, and chronic systemic disorders.

### Ophthalmic examination and AS-OCT imaging

Participants underwent comprehensive ophthalmic examinations, including slit-lamp microscopy, refractive status, intraocular pressure, fundoscopy, and endothelial cell density measurement. WTW, central corneal thickness (CCT), and corneal tomography were obtained using a Scheimpflug camera (Pentacam HR; Oculus Optikgeräte GmbH). Axial length (AL) was measured with the IOL Master 700 (Carl Zeiss Meditec AG).

**Fig. 1:**
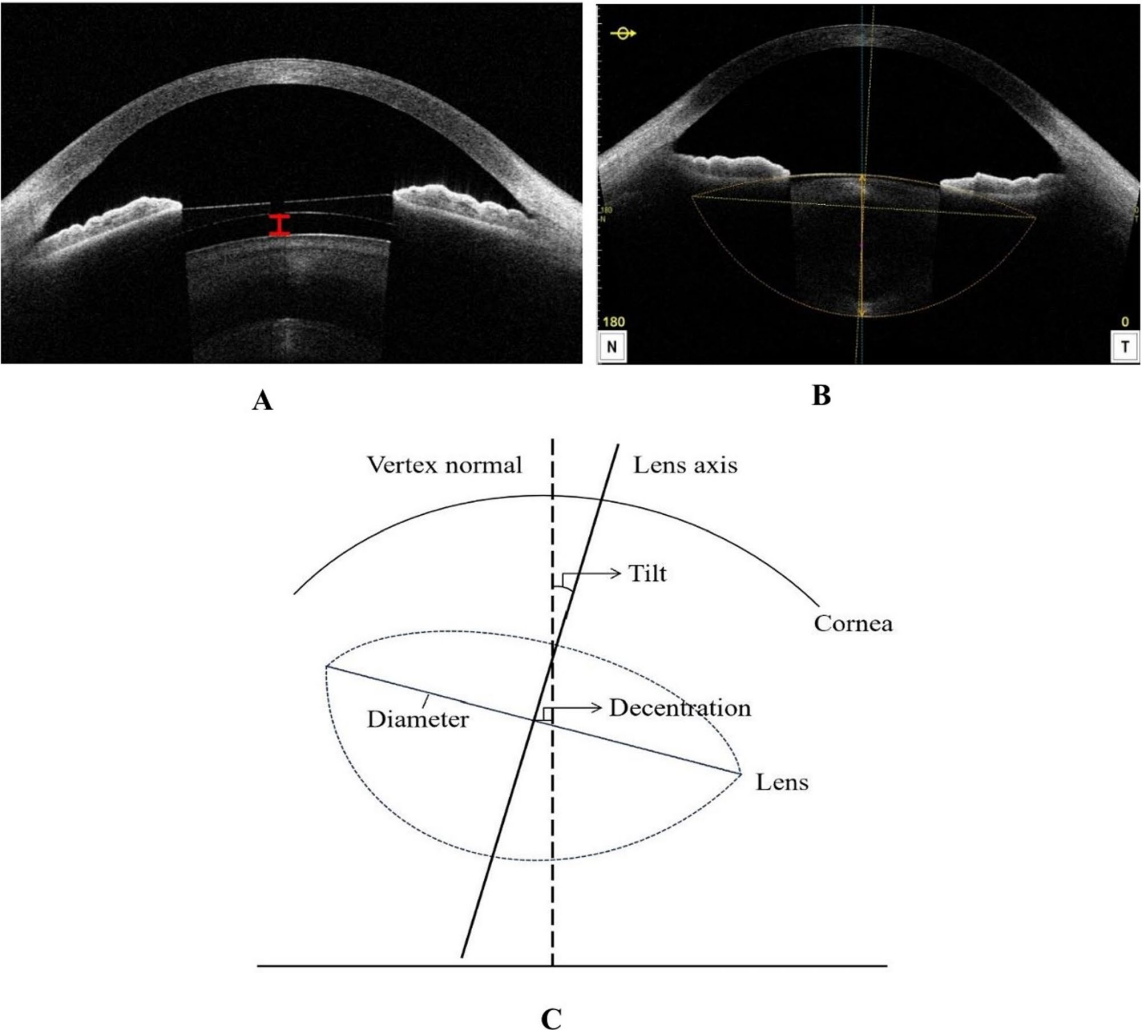
AS-OCT image showing the ICL vault and the morphology of the crystalline lens. **A** The representative image of ICL vault (vertical red solid line) obtained from the CASIA 2. **B** The representative image of anterior segment obtained from the CASIA 2. Lens axis of lens (vertical yellow dot line), vertex normal (vertical blue dot line), lens thickness (vertical orange solid line) and anterior and posterior lens boundary (orange dot lines) are automatically drawn. **C** The schematic diagram showing the measurement of crystalline lens parameters (decentration, diameter and tilt)

The right eye of each subject was scanned with AS-OCT (CASIA2, Tomey Corporation, Nagoya, Japan) by two experienced operators who were blinded to the preceding clinical data. The AS-OCT measurements were performed using the 16-scan lens biometry mode, which produces 16 Distinct two-dimensional images from Different angles. Several studies have demonstrated the high repeatability of CASIA 2 in obtaining lens parameters [12, 17]. The crystalline lens parameters, including anterior radius of curvature (ARC), posterior radius of curvature (PRC), lens thickness (LT), Diameter, decentration, and lens tilt angle, were automatically acquired by CASIA 2 built-in software (Version 3E.22) in non-dilated eyes. The best quality image among three captures was selected for analysis. All images were processed by a single experienced examiner who was blinded to clinical data. As shown in Fig. 1B, ARC and PRC represent the anterior and posterior radius of curvature of the lens (orange dotted lines), respectively. LT is the lens thickness along the vertex normal (vertical orange solid line). Lens diameter is defined as the diameter of the equatorial part of the crystalline lens (horizontal yellow dotted line). Lens decentration is defined as the vertical distance from the lens center to the vertex normal (Fig. 1C). The tilt angle is defined as the angle between the lens axis and the vertex normal (Fig. 1C). The vertex normal is defined as the line between the fixation point and the vertex of the corneal topographic map. Preoperative ACD (measured from the corneal endothelium at the corneal apex to the anterior surface of the crystalline lens) and postoperative ICL vault (3 months after surgery, Fig. 1A) were also assessed by AS-OCT.

### Statistical analysis

Statistical analyses were performed using SPSS Statistics (version 27, IBM Corp., NY, USA). The normal distribution of the data was tested using the Shapiro–Wilk test. The ANOVA test and the Kruskal–Wallis *H* test were used to compare differences among the three groups according to data distribution. The chi-square test was used to compare categorical variables. Spearman correlation analysis was conducted to evaluate the relationships between preoperative biometric parameters and vault. Multiple logistic regression analysis was performed to identify variables relevant to low vault (< 250 µm) and high vault (> 750 µm). A *P*-value < 0.05 indicated a statistically significant difference.

## Results

The demographic and clinical characteristics of patients and different vault groups are summarised in Table 1. The study cohort comprised 199 eyes from 199 participants (57 males and 142 females) with a mean age of 29.0 (10.0) years. The mean vault, measured 3 months after ICL implantation, was 444 (261) µm. Of all eyes included, 164 eyes were identified with optimal vault (OV group), 24 eyes with low vault (LV group), and 11 eyes with high vault (HV group). Clinical parameters were compared across the different vault groups. There were no significant differences among the groups except for WTW, ACD, ARC, PRC, LT, and decentration. Among parameters related to the crystalline lens characteristics measured by AS-OCT, eyes in the LV group had a steeper ARC, PRC, and thicker LT compared with the OV group (all P < 0.001), while the HV group had flatter ARC, PRC, and thinner LT compared with the OV group (all *P* < 0.001). Eyes in the LV group and HV group had greater decentration compared with the OV group (*P* < 0.001).

**Table 1:** Demographic and clinical characteristics of the study participants

	**All** **(*n* = 199)**	**LV (< 250 µm)** **(*n* = 24)**	**OV (250 µm to 750 µm)** **(*n* = 164)**	**HV (> 750 µm)** **(*n* = 11)**	***P***
Demographic and clinical parameters					
Age (years)	29.0 (10.0)	29.0 (9.75)	29.0 (10.0)	26.0 (8.00)	0.85
Gender (M/F)	57/142	5/19	50/114	2/9	0.79
SE (D)	−10.0 (4.25)	−10.88 (3.94)	−10.0 (4.50)	−7.25 (3.50)	0.22
WTW (mm)	11.6 (0.60)	11.4 (1.08)	11.6 (0.44)	12.0 (0.60)	< 0.001^**^
ACD (mm)	3.19 (0.35)	3.02 (0.22)	3.20 (0.33)	3.48 (0.33)	< 0.001^**^
CCT (mm)	0.52 (0.04)	0.53 (0.04)	0.52 (0.04)	0.54 (0.05)	0.34
Kf (D)	42.9 (1.80)	43.2 (1.06)	42.80 (1.80)	42.7 (2.20)	0.70
Ks (D)	44.2 (2.00)	44.1 (1.69)	44.20 (2.08)	44.0 (2.98)	0.68
AL (mm)	27.3 (2.20)	27.5 (2.00)	27.3 (2.38)	26.74 (1.83)	0.62
Vault (µm)	444 (261)	209 (74.8)	465 (196)	848 (104)	< 0.001^**^
Crystalline lens characteristics					
ARC (mm)	11.0 (1.06)	9.47 (2.65)	11.0 (0.92)	12.0 (0.50)	< 0.001^**^
PRC (mm)	5.88 (0.59)	5.58 (0.60)	5.90 (0.58)	6.06 (0.66)	0.01^*^
LT (mm)	3.81 (0.41)	4.22 (0.51)	3.80 (0.37)	3.77 (0.22)	< 0.001^**^
Diameter (mm)	9.71 (0.48)	9.43 (0.63)	9.74 (0.45)	9.90 (0.52)	0.08
Decentration (mm)	0.22 (0.13)	0.32 (0.14)	0.20 (0.13)	0.30 (0.10)	< 0.001^**^
Tilt (°)	3.10 (1.80)	2.65 (1.65)	3.20 (1.70)	2.50 (1.50)	0.08

Values presented as median (interquartile range, IQR)

*Abbreviations:LV* low vault, *OV* optimal vault, *HV* high vault, *SE* spherical equivalent, *D* diopters, *WTW* horizontal white-to-white diameter, *ACD* anterior chamber depth, *CCT* central corneal thickness, *Kf* flat keratometry, *Ks* steep keratometry, *AL* axial length, *ARC* anterior radius of curvature of the lens, *PRC* posterior radius of curvature of the lens, *LT* lens thickness

^*^P < 0.05; ^**^P < 0.001

**Fig. 2:**
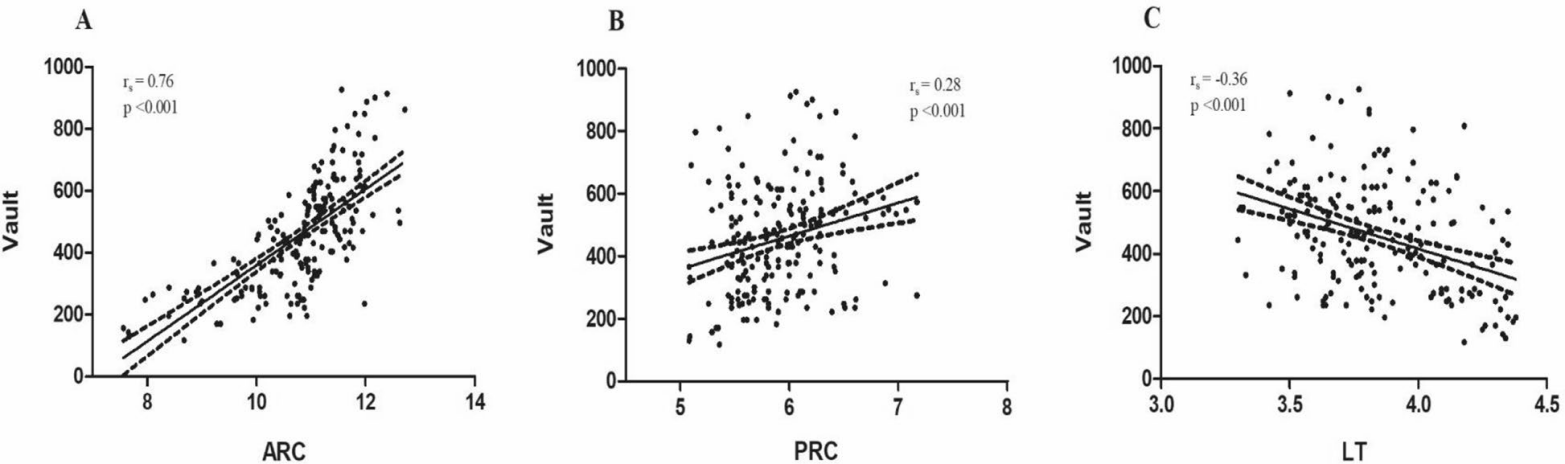
Linear correlations between postoperative vault and crystalline lens parameters, including (**A**) anterior radius of curvature (ARC), (**B**) posterior radius of curvature (PRC), and (**C**) lens thickness (LT). Linear correlation coefficient (rs) and *P* value were presented in scatter graphs

Spearman analysis was conducted to assess the correlation of other factors related to lens parameters (Table 2). The results showed that ARC, PRC, LT and diameter were the relevant lens parameters related to the postoperative vault (all *P* < 0.001). All lens parameters were significantly associated with SE. ARC was significantly associated with age, WTW, ACD and AL (all *P* < 0.05). LT was significantly associated with age, ACD and AL (all *P* < 0.05). Diameter and PRC were significantly associated with ACD (*P* < 0.001). Tilt was significantly associated with flat keratometry and AL (all *P* < 0.05). No correlation was found between lens parameters and gender or steep keratometry. Additionally, there was a linear relationship between vault and factors related to the crystalline lens parameters (Fig. 2). The postoperative vault was positively correlated with ARC (rs = 0.76, *P* < 0.001) and PRC (rs = 0.28, *P* < 0.001), and negatively correlated with LT (rs = −0.36, *P* < 0.001).

**Table 2:** The association of lens parameters by spearman analysis

	**ARC**		**PRC**		**LT**		**Diameter**		**Decentration**		**sTilt**	
	**r**	***P***	**r**	***P***	**r**	***P***	**r**	***P***	**r**	***P***	**r**	***P***
Vault (µm)	0.76	< 0.001^**^	0.28	< 0.001^**^	−0.36	< 0.001^**^	0.27	< 0.001	0.04	0.62	0.06	0.42
Age (years)	−0.19	0.01^*^	−0.06	0.41	0.48	< 0.001^**^	0.03	0.64	−0.05	0.49	−0.11	0.14
Gender (M/F)	0.06	0.42	0.06	0.42	−0.07	0.35	−0.04	0.58	0.05	0.50	0.09	0.21
SE (D)	0.49	< 0.001^**^	0.24	< 0.001^**^	−0.34	< 0.001^**^	0.24	< 0.001^**^	0.16	0.02^*^	0.17	0.01^*^
WTW (mm)	0.27	< 0.001^**^	0.14	0.05	−0.17	0.02^*^	0.11	0.13	−0.07	0.33	0.03	0.64
ACD (mm)	0.47	< 0.001^**^	0.51	< 0.001^**^	−0.43	< 0.001^**^	0.42	< 0.001^**^	0.03	0.67	0.14	0.04^*^
Kf (D)	−0.02	0.77	−0.02	0.80	0.01	0.90	−0.11	0.13	−0.09	0.21	0.16	0.03^*^
Ks (D)	0.03	0.66	0.06	0.43	−0.02	0.78	−0.003	0.96	−0.08	0.27	0.11	0.14
AL (mm)	−0.33	< 0.001^**^	−0.12	0.11	0.22	0.002^*^	−0.07	0.34	−0.08	0.26	−0.20	0.004^*^

*SE* spherical equivalent, *D* diopters, *WTW* horizontal white-to-white diameter, *ACD* anterior chamber depth, *CCT* central corneal thickness, *Kf* flat keratometry, *Ks* steep keratometry, *ARC* anterior radius of curvature of the lens, *PRC* posterior radius of curvature of the lens, *LT* lens thickness, *AL* axial length

^*^P < 0.05; ^**^P < 0.001

Multiple logistic regression analysis used the preoperative biometric parameters (including WTW, ACD, AL, and crystalline lens parameters) to determine the influence of relevant variables on low vault and high vault (Table 3). The results revealed that ARC and tilt were associated with low vaults after ICL implantation (*P* < 0.05).

**Table 3:** Multiple logistic regression analysis assessing the factors related to sub-optimal vault (< 250 µm, > 750 µm) (*n* = 199)

Variable	Low vault				High vault			
	***β***_**L**_	**SE**	**OR**	**OR 95%CI**	***β***_**H**_	**SE**	**OR**	**OR 95%CI**
WTW (mm)	−0.13	0.60	0.83	0.27 to 2.85	1.11	0.99	3.04	0.44 to 21.15
ACD (mm)	−0.33	0.75	0.72	0.17 to 3.13	2.11	1.34	8.26	0.60 to 113.22
AL (mm)	−0.83	1.14	0.44	0.05 to 4.10	−0.72	1.31	0.49	0.04 to 6.37
ARC (mm)	−4.10	1.23	0.02	0.002 to 0.18^**^	1.85	1.29	6.36	0.51 to 79.34
PRC (mm)	1.29	0.99	3.62	0.52 to 25.34	1.54	1.15	4.65	0.48 to 44.63
LT (mm)	1.45	0.76	4.25	0.96 to 18.89	0.51	0.91	1.66	0.28 to 9.96
Diameter (mm)	−0.83	0.89	0.44	0.08 to 2.52	−1.05	1.11	0.35	0.04 to 3.09
Decentration (mm)	1.28	0.65	3.58	1.01 to 12.67	1.61	0.91	5.01	0.85 to 29.52
Tilt (°)	−1.55	0.65	0.21	0.06 to 0.75^*^	−0.60	0.85	0.55	0.10 to 2.92

*β* regression coefficient (*β*_L_ and *β*_H_: regression coefficients for low and high vault), reference group: optimal vault (*n* = 164)

*SE* standard error, *OR* odds ratio, *CI* confidence interval, *WTW* horizontal white-to-white diameter, *ACD* anterior chamber depth, *AL* axial length, *ARC* anterior radius of curvature of the lens, *PRC* posterior radius of curvature of the lens, *LT* lens thickness

^*^P < 0.05; ^**^P < 0.001

## Discussion

According to the latest consensus statement, AS-OCT demonstrates excellent intra- and inter-examiner reproducibility in the measurement of anterior segment parameters [18]. In the present study, we utilized AS-OCT to further investigate the influence of biometric and detailed crystalline lens characteristic parameters associated with postoperative vaults in Chinese patients with high myopia. The results demonstrated that the ARC, PRC, LT and diameter were the relevant lens parameters related to the postoperative vault. In addition, we also found that the ARC and tilt of the crystalline lens were morphological features correlated with low vaults after ICL implantation.

The lens shape varies throughout refractive development from low myopia to high myopia [19]. Meanwhile, the morphological changes of the crystalline lens have been a pivotal issue in myopia progression [12]. Thus, lens changes induced by high myopia may statically affect anterior segment biometry. Historically, both the shape of the crystalline lens and relative lens position have been deemed influential over postoperative vault value. Qi MY et al [15] demonstrated a positive correlation between vault and preoperative ACD as well as lens position, while revealing a negative correlation with lens thickness. A recent study [20] investigated factors related to insufficient vault and indicated that eyes with a thicker lens and shorter AL had a significant correlation with insufficient postoperative vault. Our findings are in agreement with previous studies describing that a forward lens position or thicker LT could result in lower postoperative vault.

The anterior lens surface position, representing the distance between the sulcus-to-sulcus plane and the anterior crystalline lens surface (STSL), was considered a key predictor for optimal ICL sizing [14, 21]. High STSL and low SE are major risk factors in eyes presenting low postoperative vaults [22]. A previous study by Zheng et al [14] found ARC to be one of the major factors determining the STSL value and potentially a better predictor of postoperative vault than STSL. In the present study, we observed that a steeper ARC was found in the LV group, whereas the HV group tended to have a significantly flatter ARC. However, we found no correlation between decentration and postoperative vault by Spearman correlation test. This finding might be related to the same degree of myopia chosen in this study. In this condition, decentration demonstrated no significant changes and thus had less effect on the postoperative vault. Additionally, our study showed that ARC and tilt were significantly associated with sub-optimal vaults. The origin of the zonule starts at the ciliary body tips and extends to the equatorial segment of the crystalline lens. Considering that ARC and lens tilt partially rely on the tension of the zonules, we speculated that highly myopic eyes with different zonular tensions might lead to different postoperative vaults. Additionally, a study proposed a positive correlation between crystalline lens shape and AL [23]. Other factors contributing to sub-optimal vault include the relatively smaller ICL sizing implanted in these eyes and possible AL-related zonular weakness. In highly myopic eyes, progressive axial elongation induces mechanical stretching of zonular fibers, thereby reducing their baseline tension [24]. This zonular laxity contributes to irregular lens tilt [25], and greater tilt magnitude causes asymmetric contact points between the posterior ICL surface and the anterior lens capsule. Such asymmetry creates uneven support forces that can predispose the vault to inferior displacement [24, 25]. Our findings align with existing literature showing that zonular instability affects lenticular positioning, confirming zonular integrity as a critical determinant of postoperative anatomy. These biomechanical interactions suggest that crystalline lens morphology is not only an anatomical indicator of zonular health, but also a key predictor of vault configuration.

The present study was limited by its retrospective design, particularly by the small number of participants in high vault groups. Consequently, results for high vault eyes are exploratory, and the distribution of the lens morphological parameters in these eyes may not accurately represent the general situation. Additionally, the lens morphological parameters in our study were calculated using formulas provided by inbuilt semi-automated software, which could potentially introduce bias. Direct measurements from complete imaging of the lens might provide more comprehensive information. Furthermore, our study focused solely on high myopia, and the absence of records for low-to-moderate myopia might limit our understanding of their roles in affecting postoperative vault. A large prospective cohort study encompassing different types of refractive errors is essential to confirm our findings in the future, and establish evidence-based thresholds that can reliably guide clinical decisions.

In conclusion, lens parameters are crucial for predicting postoperative vault. Our study also demonstrated that high myopic patients with low vaults exhibited significantly altered ARC and lens tilt. These findings suggest that ARC and lens tilt may serve as additional anatomical characteristics in eyes with low vaults following ICL implantation.

## Data Availability

The datasets used and/or analyzed during the current study are available from the corresponding author on reasonable request.
